# Australian University Students’ Experience of Animal-Assisted Education: An Exploratory Study

**DOI:** 10.3390/ani14192792

**Published:** 2024-09-27

**Authors:** Jessica Hill, Lucy Waldby, Teresa Quinlan, Jennifer Fleming, Melanie Hoyle, Carlie Driscoll

**Affiliations:** School of Health and Rehabilitation Sciences, The University of Queensland, Brisbane, QLD 4072, Australia; jessica.hill@uq.edu.au (J.H.); l.waldby@uq.edu.au (L.W.); t.quinlan@uq.edu.au (T.Q.); j.fleming@uq.edu.au (J.F.); m.hoyle@uq.edu.au (M.H.)

**Keywords:** animal-assisted education, animal-assisted interventions, mental health, student wellbeing, student engagement

## Abstract

**Simple Summary:**

University students frequently experience poor mental health. Whilst many universities have made it a priority to provide services directed towards supporting student mental health and wellbeing, several barriers to accessing these services exist, reducing their overall uptake. Animal-assisted education implemented within primary and secondary schools has shown promise as a potential intervention to support student mental health and wellbeing. Furthermore, while some studies have evaluated the impact of animal-assisted activities in university settings, there is a specific gap in the research concerning animal-assisted education. This study aimed to gain a preliminary understanding of the experience of university students involved in animal-assisted education. Findings showed that the students valued the presence of the therapy dog within their learning activities. They particularly highlighted the benefits they experienced in their overall mood, learning experiences, and social engagement. This study highlights the need for further research into the influence of animal-assisted education on student mental health and wellbeing.

**Abstract:**

University students experience poorer mental health outcomes when compared to the general population. Poor mental health has been associated with reduced wellbeing and low academic performance, resulting in higher rates of withdrawal. Animal-assisted education is an intervention found to result in a reduction in anxiety and an increased learning engagement among primary and secondary students. However, minimal research has been conducted regarding the inclusion of therapy dogs in the learning environments of students in tertiary education. This study explored the influence of animal-assisted education on the experience of university students. A total of 56 university students engaged with animal-assisted education over a 13-week period and completed an online survey comprised of open- and closed-response questions. The findings showed that all perceived benefits of the therapy dog, including improved mood and reduction of anxiety, increased motivation and engagement in learning activities, as well as an improved social engagement with peers and the educator. Preliminary findings demonstrated that the inclusion of animal-assisted education into the teaching of university students may assist in supporting their mental health and overall learning experience. Research is needed to explore the most effective ways to incorporate animal-assisted education into university settings for both students and therapy dogs.

## 1. Introduction

The mental health of university students has been recognised as an important public health issue on a global level [1]. On average, university students experience poorer mental health when compared to the general population due to the challenges associated with the developmental transition of being an emerging adult, as well as the stressors that often result from tertiary education, such as course work demands and assessments [2,3]. Poor mental health has been correlated with having a negative impact on the overall health and wellbeing of students, as well as placing them at risk of poor academic performance and higher rates of withdrawal [3].

Acknowledging these statistics, supporting the mental health and wellbeing of university students has become a priority for many Australian universities, resulting in the development of several wellness programs [2,3]. Despite these efforts, it has been suggested that young Australians experiencing poor mental health may be reluctant to engage in help-seeking behaviours such as accessing these services [2]. For university students specifically, it has been estimated that as little as 22% of students at risk of poor mental health seek out help through formal university student wellbeing initiatives [4]. Potential barriers to accessing these supports involve students’ own attitudes towards the available programs (e.g., anticipated benefits vs. time demands), self-stigma of accessing such programs, and their own self-awareness of need [2].

Within primary and secondary schools, animal-assisted education has gained increasing attention as a method of supporting student mental health and wellbeing [5,6]. Positioned under the umbrella term of animal-assisted interventions (also including animal-assisted activities and animal-assisted therapy), animal-assisted education involves a trained and assessed animal that is purposely incorporated into structured, goal-directed activities or programs with educational outcomes [6]. Although several species have been included into animal-assisted education, due to their unique ability to respond to human verbal and non-verbal cues, dogs (referred to as therapy dogs for the remainder of this article) are the most frequently incorporated species [5,7]. Research within primary and secondary schools has found that including a therapy dog into student learning activities can support attention and learning engagement, as well as social and emotional development and psychological wellbeing [5,7].

Several universities have also begun to explore how animal-assisted interventions might be incorporated to support student mental health and wellbeing, as well as learning engagement [8,9,10]. However, within universities, these programs have typically involved visitation animals delivering animal-assisted activities. Different to animal-assisted education, animal-assisted activities involve a human handler who volunteers their time to visit facilities with their visitation animal to deliver animal interactions and activities with the goal of bringing enjoyment [6]. Within the context of universities, animals have been brought onto campus by their handler to provide ‘animal-assisted events’ [10], ‘therapy dog interactions’ [9], or ‘therapy dog programs’ [8]. Whilst all differing slightly in their implementation (e.g., frequency, duration, activities involved, etc.), the benefits of these programs reported by the students have included improved mood and satisfaction of campus life, improved academic success, the uptake of stress prevention programs, and a reduction in homesickness and stress [8,9,10,11,12,13]. Although these results are promising, the availability of animal-assisted activities delivered on university campuses does not remove the barrier of students first needing to be aware of these opportunities, as well as requiring the initiative to actively seek them out.

In contrast to visitations, animal-assisted education involves the purposeful inclusion of therapy dogs into learning activities [6]. As such, having the therapy dog included into learning activities for all students has the potential to remove the barrier of students needing to independently seek and access these services. To the authors’ knowledge, limited research has been conducted exploring the influence of animal-assisted education on the mental health and wellbeing, and learning engagement of university students [14,15]. As such, this exploratory study aimed to gain a preliminary understanding of the experience of university students involved in animal-assisted education.

## 2. Methods

### 2.1. Animal-Assisted Education

This study was conducted at The University of Queensland, Brisbane, Australia, with students completing a Bachelor or Graduate Entry Masters in Occupational Therapy. Animal-assisted education sessions were delivered across a 13-week period, within the first semester of the students’ second year of study [16]. The therapy dog involved was an eight-year-old female standard labradoodle who had completed training and assessment as a therapy dog and had seven years’ experience working with children and adult populations. The educator/therapy dog handler delivering the animal-assisted education was a registered occupational therapist with additional training in animal-assisted interventions. Further, she was appointed at The University of Queensland as a teaching and research academic. The therapy dog attended weekly, 90-min face-to-face tutorials delivered by the educator who was also the therapy dog’s owner and handler. The course chosen was designed to develop student knowledge and practical skills when working with children with developmental disabilities such as autism [16]. Due to the increased interest in research and in practice of the benefits of animal-assisted therapy with autistic children, this course was deemed most appropriate [16].

Within each session, the therapy dog was actively incorporated into a minimum of one teaching activity. Examples of such activities include the educator demonstrating a client intervention whilst incorporating the therapy dog, as well as the students having the opportunity to work with the therapy dog under the supervision of the educator. During the remainder of the learning activities, the therapy dog was present in the room and was off-lead, allowing her to interact with the students and rest on her own volition. The students were made aware that they were able to interact with the therapy dog throughout the learning activities, with interactions involving watching, talking to, physical touch (e.g., patting), and playing with her toy. Water was always made available to the therapy dog, and toilet breaks occurred before and after each session.

Prior to the inclusion of the therapy dog, students were also made aware that if they did not wish to interact with the therapy dog (e.g., allergies, fear of dogs, etc.), they could contact the educator to discuss solutions to ensure their learning experience within the course was not impacted. No students approached the educator to express that they did not wish to interact with the therapy dog.

### 2.2. Questionnaire Development

As there were no pre-existing measures available, a questionnaire titled ‘Animal-assisted education for university students: A survey study’ was custom-designed based on an extensive review of the literature [9,10,11,12,13], as well as the expertise of the first author (JH—mental health occupational therapist, and teaching and research academic with ten years of clinical and seven years of research experience in animal-assisted interventions). Part one of the survey collected demographic data using closed-response questions. Part two of the survey aimed to understand how the students perceived their mental health and wellbeing in relation to their university studies. Using a five-point Likert scale, participants were asked to rate the frequency (1 = never/almost never, 5 = daily) with which they experienced academic, financial, social, and family stress, social isolation, homesickness, as well as feelings of depression and anxiety. Finally, part three of the survey explored participants’ overall experience of animal-assisted education through closed- and open-response questions. Within each closed-response question, participants were asked to respond to each item using a five-point Likert scale, with one indicating lower levels of enjoyment/agreement and five indicating higher levels of enjoyment/agreement. Examples of questions featured were “How did you enjoy the learning activities with the therapy dog present?” and “Do you feel your mood was influenced by the presence of the therapy dog?” Students were also provided with three additional open-response questions, including (1) “How would you describe your overall experience of animal-assisted education?” (2) “What were the benefits (if any) of including a therapy dog into student learning activities?” (3) “What recommendations would you make for future animal-assisted education programs at university?” A draft of the survey tool was pilot-tested, before wider distribution, by one independent examiner regarding its usability and relevance of questions. This involved the examiner being provided with a hyperlink of the survey tool to complete. Written feedback was provided to the research team for each question. The examiner was an occupational therapist with experience in teaching and survey design. The draft survey tool was reviewed and modified by JH prior to commencing data collection.

### 2.3. Data Collection

Using a convenience sample, students were eligible to complete the survey if they were occupational therapy students at The University of Queensland and had attended tutorials involving the therapy dog. Surveys were distributed at the end of the 13-week semester through the online survey software Qualtrics [Version 2022], allowing for data to be collected anonymously. Eligible students were emailed directly and were required to provide informed consent prior to commencing the survey. Students were informed that participation in the survey was voluntary and anonymous, and that their responses, or lack thereof, would not affect their course progression.

### 2.4. Data Analysis

Deidentified quantitative data were downloaded and analysed through Microsoft Excel [Version 16.63.1]. Each question was examined using frequencies of responses and converted into percentages of total responses. Qualitative data were analysed through content analysis to identify common words, ideas, and categories [17]. All open-answer responses were initially coded by the first author (JH) following a manifest analysis methodology outlined by Bengtsson [17]. Within this methodology, the author describes four stages. In stage one, decontextualization data familiarisation occurred, in which responses to each open-response question were read, and preliminary indicative codes were generated. In stage two, recontextualization of all codes generated was considered in relation to their relevance to the overall aim of the research. In stage three, categorisation occurred, in which preliminary categories were identified. Finally, in stage four, the compilation findings were reported in line with the specific quotes provided by participants. To enhance the validity and reliability of the final findings, this four-stage process was shared with author CD, who reviewed all codes and final categories [17].

## 3. Findings

### 3.1. Participant Demographics

A total of 56 occupational therapy students completed the survey, with participant ages ranging from 18 to 42 years (M = 23.29, SD = 6.69). The majority were female (*n* = 49, 87.5%), and most were employed at least casually while studying full-time (*n* = 40, 70.4%). A little over half (*n* = 28; 50.9%) of participants lived within the family home, and almost all (*n* = 49, 87.5%) had experience owning a pet, with many (*n* = 26, 46.4%) owning a dog. A full description of participant demographics can be found in Table 1.

**Figure 1:**
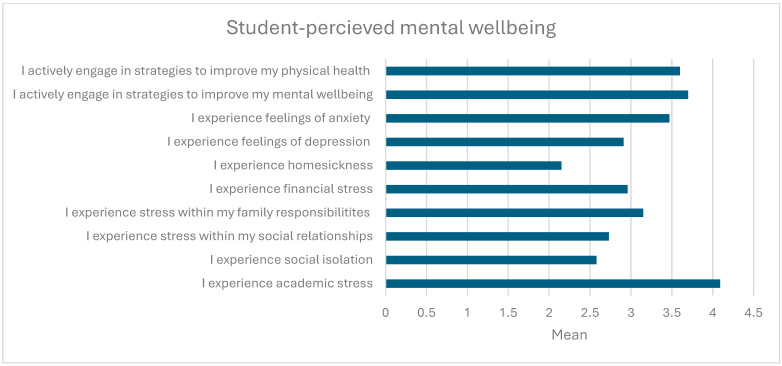
Student-perceived mental wellbeing rated on a five-point scale (N = 56).

Figure 1 shows participants’ ratings of their perceived mental health and wellbeing on a five-point Likert scale from one (never/almost never) to five (frequently/daily). Overall, participants indicated experiencing a moderate level of stress across all determinants; however, they rated their academic stress highest (M = 4.09, SD = 0.69). On average, students also reported occasionally (i.e., monthly) engaging in strategies to support their mental (M = 3.7, SD = 1.00) and physical (M = 3.6, SD = 1.8) health. When asked to rate whether they agreed or disagreed (1 = strongly disagree, 5 = strongly agree) that their university had adequate services to support their mental health and wellbeing, on average, students indicated moderate agreement (M = 3.22, SD = 1.01) that their university provided adequate mental health support services.

### 3.2. Experience of Animal-Assisted Education

#### 3.2.1. Quantitative Data

Table 2 summarises participants’ responses to questions about their experience of animal-assisted education. Almost all participants reported enjoying animal-assisted education (*n* = 53, 92.9%) and would recommend that other universities include animal-assisted education in learning activities (*n* = 53, 98.1%). Participants indicated that they engaged with the therapy dog in a range of different ways, with most identifying physical touch such as patting and hugging (*n* = 51, 91%), watching (*n* = 42, 75%), and talking to the therapy dog (*n* = 31, 55.4%). Most participants perceived the inclusion of the therapy dog improved their overall learning experience (*n* = 38, 67.9%), including changes in mood (*n* = 47, 83.9%), motivation (*n* = 39, 69.6%), and engagement in learning activities (*n* = 34, 60.7%). Overall, participants saw value in including a therapy dog in learning activities throughout the whole semester.

#### 3.2.2. Qualitative Data

The first open-response question asked participants to describe their overall experience of animal-assisted education, with 46 (81.2%) students providing a response. Responses fell under six categories: (1) overall positive experience (*n* = 22, 47.8%), (2) positive influence on mood (*n* = 17, 37%), (3) supportive of motivation (*n* = 8, 17.4%), (4) facilitated social relationships (*n* = 4, 8.7%), (5) a beneficial learning opportunity (*n* = 2, 4.3%), and (6) potential challenges (*n* = 2, 4.3%). Under the category of overall positive experience, many participants described enjoying their experience of animal-assisted education using words such as “enjoyable”, “amazing”, “delightful”, “wonderful”, and “beneficial”. Several participants also advocated for increased opportunities for animal-assisted education to be included in university courses, with one student highlighting this view by stating, “I really enjoyed it and would like to see it more”.

Within category 2, positive influence in mood, participants described ways in which the inclusion of the therapy dog in learning activities assisted in supporting their overall mood. Specifically, participants described how the therapy dog created a more “relaxed” environment, providing “comfort” and a “calming presence” during class. Several participants specifically identified that the therapy dog assisted in supporting their overall mental health, with one participant describing, “I struggle with various mental illnesses and [the therapy dog’s] presence has been extremely comforting and benefits my mental health”. Similarly, a second participant explained that they “enjoyed it thoroughly, and [animal-assisted education] really calmed me down in stressful periods of school life”.

Participants also discussed how animal-assisted education aided their learning. In category 3, supportive of motivation, participants described how the therapy dog supported their motivation to attend class and engage with the learning activities. For example, one participant reported, “I can see how it motivated a lot of the students to attend class and understand the content better”. A second participant further highlighted the therapy dog’s influence on their motivation by explaining, “I really enjoyed the presence of the therapy dog in class. It kept me extremely motivated to attend class—I have never missed a single [class] when [the therapy dog] was present”. In category 4, participants also discussed how the inclusion of the therapy dog facilitated social relationships with both their peers and the educator. These sentiments were highlighted by two participants who stated, “the presence of the therapy dog helped me establish connections with not only my peers but also my educator”, and “[the therapy dog] also enabled me to interact with other students that I barely knew. She provided a social glue between us”. As the students included in this research were studying occupational therapy, within category 5, participants also described how the educator was able to incorporate the therapy dog as a teaching opportunity to demonstrate how animal-assisted therapy could be included in occupational therapy practice. For example, one participant explained, “[the therapy dog] was an excellent learning aid when being taught about the inclusion of animal-assisted therapy”.

Despite the positives of animal-assisted education discussed by many of the participants, within category 6, two students also highlighted potential challenges. Specifically, these participants identified that if not implemented correctly, there was potential for the therapy dog to act as a “distraction”, particularly for students who experience visual and auditory sensitivity.

The second open-response question asked participants to describe any potential benefits of including a therapy dog into student learning activities. A total of 36 participants (64.3%) responded to this question, with responses falling into four categories: (1) providing comfort and support (*n* = 19, 52.8%), (2) enhanced motivation to learn (*n* = 14, 41.7%), (3) strengthening social relationships (*n* = 11, 30.6%), and (4) a beneficial teaching strategy (*n* = 9, 25%). Within category 1, providing comfort and support, participants described that the “comfort” and “stress relief” the presence of the therapy dog brought to students during learning activities. One participant provided details on a specific challenging time in her life in which the therapy dog brought her emotional support:

“One day at uni I was having a personal crisis and was feeling extremely anxious and I wanted to go home but getting the opportunity to pat and connect with [therapy dog] made a huge difference for my mental state that day and helped me regulate my emotions and I was able to stay at uni for the day”.

In category 2, it was again noted by participants that animal-assisted education enhanced motivation to learn. Within this category, several students described how knowing the therapy dog would be present motivated them to attend class in the first instance, which ultimately aided their learning outcomes. For example, one participant explained:

“Knowing the therapy dog would be at a tutorial was very motivating for me to attend, especially considering that my tutorial with the dog was the only class I had on at uni that day and knowing the dog would be there made me less likely to skip the tutorial…For me I’ve always found that the more I participate and attend learning activities the better I understand the content, therefore knowing the dog would be there ultimately lead to better learning outcomes for me”.

For other students, they believed that the inclusion of the therapy dog aided their “engagement” and “attention” in the learning activities, with two students explaining, “helped me engage with class and concentrate during down time,” and “it helps students pay better attention in class”.

Within category 3, participants reiterated how the support provided by the therapy dog helped build and strengthen social relationships. Several participants highlighted the positive influence that dogs could have in challenging social situations. For example, one participant explained, “it brought us together as a group, I think. It softened the social harshness of the classroom, and reduced barriers to communication between students”. Another described this impact by explaining, “dogs seem to have a positive effect on the class. It was energising to have her around, and a conversation starter when we were getting to know the course content/other students”. Several students again also highlighted that the presence of the dog supported the development of their relationship with their educator, with one explaining, “it made it easier to connect with the [educator] which made it less stressful to ask for support”.

In category 4, occupational therapy students further described the inclusion of the therapy dog as a beneficial learning strategy. Specifically, participants noted that the practical demonstrations performed by the educator assisted in aiding their understanding of what animal-assisted therapy could look like in practice. One participant highlighted this point by explaining that it “allowed us to see how animal-assisted therapy can occur within clinic sessions,” whilst another reported, “at times the therapy dog could even be incorporated into the class material, which aided learning and understanding and even helped with remembering the content”.

Finally, the third open-response question asked participants what recommendations they had for future animal-assisted education programs implemented within university settings. A total of 29 (51.8%) participants responded to this question, with answers falling within four categories: (1) increased implementation of animal-assisted education within universities (*n* = 15, 51.7%), (2) increased inclusion of therapy dogs within universities in general (*n* = 6, 20.7%), (3) increased hands-on experience of animal-assisted therapy (*n* = 5, 17.2%), (4) no further recommendations (*n* = 2, 6.9%).

Within category 1, students described the benefits they had gained from animal-assisted education and advocated for other courses to implement this strategy. This was particularly true for courses that were considered by students to be more “stressful”. For example, one participant explained:

“I would really enjoy having a therapy dog in some of our more difficult subjects like anatomy or physiology, as these subjects are very stressful, and I left the classes feeling quite defeated more than once. Even in the difficult class that had the therapy dog I always left the tutorial feeling happy and refreshed”.

Within category 2, several participants perceived that additional opportunities to engage with therapy dogs, outside of learning activities, would also be beneficial. Two students provided specific examples of this, with one identifying the potential for “a small session where students can play with the [therapy dog] after doing tutorial content”, and another advocating for “more opportunities for students to interact with [therapy dogs], including them more in [tutorials], uni spaces, etc”.

Within category 3, students again emphasised the benefits they gained from observing how animal-assisted therapy was delivered in practice and advocated for further education on animal-assisted therapy within the field of occupational therapy. One student emphasised this point by advocating for “more hands-on and actually seeing and feeling the benefits by having animals there to consolidate our learning of animal-assisted therapy”.

Within the final category, two students identified that they had no further recommendations on how the program could be changed or improved.

## 4. Discussion

The researchers in the present study aimed to understand the potential value of animal-assisted education in supporting the mental health and wellbeing and learning engagement of university students. Like the past literature [14,15] that evaluated the effectiveness of animal-assisted activities within universities, almost all students identified that they experienced benefits from having a therapy dog included in their learning activities. Also consistent with past research, participants in this study most highly valued the influence of a therapy dog on their overall mood when attending classes. Hunt and colleagues [18] evaluated changes to university student wellbeing after attending a therapy dog interaction and found that students experienced an improvement in overall mood as measured by reductions in stress and improvements in happiness and energy levels after the interaction. Similarly, Grajfoner et al. [19] evaluated the effect of a 20-min ‘dog-assisted intervention’ on the wellbeing and mood of 132 university students, also finding a significant improvement in student-reported mood. In the present study, participants perceived themselves to experience a moderate level of daily stress. Given this finding and the high prevalence of poor mental health among university students reported in the literature, further research is needed to thoroughly examine the changes in student mood pre- and post-intervention and determine whether changes are sustained post-class and over time. Additionally, as the benefits reported by the students in the present study are similar to those who attended animal-assisted activity sessions, additional research is needed to compare the influence of animal-assisted education versus animal-assisted activities on the overall student experience.

The second most frequently cited benefit by students was the influence of the therapy dog on motivation and engagement in the learning activities. Due to the current gap in the literature relating to the inclusion of animal-assisted education in the university context, this was a novel finding. However, the perceptions of the students involved in this study align with the past literature exploring the influence of animal-assisted education in schools. For example, a survey by Baird and colleagues [20] investigated the influence of animal-assisted education on the wellbeing of students from kindergarten to year 12. Participants included school leaders, teachers, mental health professionals, and researchers who perceived that the inclusion of therapy dogs in the learning activities of primary and secondary school students had the potential to facilitate student learning engagement [20]. Interestingly, like two participants in the present study who identified potential challenges, some participants highlighted the potential of the therapy dog to be a distraction for some students [20]. As such, further research is needed to develop guidelines and frameworks on how therapy dogs can most effectively be included in university learning activities.

Students in the present study also highlighted how the therapy dog acted as a facilitator of social interaction with their peers and educator. Although limited literature exists specifically relating to animal-assisted education in a university context, previous research relating to the social lubricant effect dogs can have both within school settings [7] and therapy sessions [21] supports this finding. For example, when purposefully included in school reading programs, therapy dogs can support the social engagement of students with their peers and teachers [7]. Similarly, when incorporated in therapy sessions, therapy dogs can help foster the development of the therapeutic relationship between the client and the therapist [21]. Considering the literature [1,2,3] on stressors faced by many university students, such as relocating to new cities, meeting new people, and forming new networks when embarking on their university careers, animal-assisted education may be worth exploring further for its potential benefits in addressing this problem.

A final novel finding of this study was the benefit that the students received from observing their educator incorporate the therapy dog directly into teaching demonstrations and role plays. This could be achieved, as the educator involved in this study was an occupational therapist and was teaching occupational therapy students about the practical application of interventions when working with child populations. In the field of occupational therapy, several researchers have advocated for the increased availability of hands-on, practical education on animal-assisted therapy to support its safe and effective implementation [22,23]. As such, incorporating therapy dogs into the learning activities of occupational therapy students may help address this need.

## 5. Limitations

This exploratory study has provided new findings relating to the potential benefits of animal-assisted education to the mental health and wellbeing of university students. However, due to its exploratory nature, several limitations should be considered. As this animal-assisted education program was a pilot program at The University of Queensland, convenience sampling was used, resulting in a small sample size. Further, as participation was voluntary, there may have been a sampling bias toward students with a preference for interacting with dogs. No specific questions were asked relating to potential challenges of animal-assisted education; however, two students did identify this potential. To ensure the experiences of all students are equally represented, further research should ensure that both benefits and challenges of animal-assisted education are sufficiently explored.

Measurement bias should also be considered. As the animal-assisted education is an emerging field, there were no pre-existing measures available to evaluate the influence of animal-assisted education on the students’ experience. Therefore, the questionnaire used was custom-designed for this study. Although based on the literature and the pilot test prior to implementation, the findings should be interpreted with this in mind. Finally, it is also important to acknowledge that self-reported measures have the potential to be subjective and therefore risk social desirability bias. Further longitudinal and cohort studies are required to further establish the potential influence of animal-assisted education on student mental health and learning engagement.

## 6. Implication for Practice and Research

Findings from this exploratory study suggest that the implementation of animal-assisted education may support the mental health and wellbeing and learning engagement of university students. Further research is required to develop clear guidelines and protocols for how therapy dogs can be included into learning activities of university students in a way that is safe and effective for the students, whilst also considering the wellbeing of the therapy dog. Whilst findings of this study highlighted potential benefits of including a therapy dog in the learning activities of university students, further rigorous research is required to explore these benefits. Examples of such research might include a randomised control trial evaluating the impact of animal-assisted education on student mental health and wellbeing outcomes, as well as education outcomes pre- and post-intervention. Examples of specific outcomes worth exploring include changes in stress and anxiety, course attendance, attitude towards learning and the educator, and social relationships with peers. Additionally, since the inclusion of animal-assisted activities in universities has shown benefits [9,10,11,12,13], research evaluating any differences between the two forms of intervention on student outcomes is recommended.

## 7. Conclusions

This pilot evaluation indicates that there are potential benefits to including a therapy dog in the learning activities of university students. Specifically, students highlighted the benefits they experienced relating to improved mood, motivation for learning activities, enhanced social engagement with their peers and the educator, as well as the benefits from observing the therapy dog being incorporated into practical examples of animal-assisted therapy. Ongoing research is required to further evaluate the extent of these benefits and to understand if improvements in mood and learning motivation are sustained outside of the animal-assisted education sessions.

## Figures and Tables

**Table 1:** Participant demographics; N = 56.

Item	N	%
Gender		
Female	49	87.5
Male	6	10.7
Gender variant/non-conforming	1	1.8
Program of study		
Undergraduate	38	67.9
Graduate entry masters	18	32.1
Previous history of university studies		
Have completed previous university degree.	39	69.6
First time studying at university.	17	30.4
Living arrangements *n* = 55		
Domestic student living within the family home.	28	50.9
Domestic student living in Queensland; however, have had to relocate to complete their studies.	10	18.2
Domestic student from interstate needing to relocate to complete their studies.	4	7.3
International student needing to relocate to complete their studies.	13	23.6
Employment		
Employed casually	28	50
Not currently employed	16	28.6
Employed part-time	9	16.1
Employed full-time	3	5.3
Previous pet history		
Yes	49	87.5
No	7	12.5

**Table 2:** University student experience of animal-assisted education (N = 56).

Item	N	%
* What type of interaction did you engage in with the therapy dog? N = 56		
Physical touch (e.g., pat, hug, etc.)	51	91
Watch	42	75
Talk to	31	55.4
As a bridge to build connection with my educator	27	48.2
Play with toy	18	32.1
As a bridge to connection with my peers	17	30.4
How did you feel about the inclusion of the therapy dog within learning activities?		
Enjoyed	52	92.9
Somewhat enjoyed	3	5.4
Neutral	1	1.9
How bonded did you feel to the therapy dog?		
Neutral, I felt the therapy dog wasn’t fazed by my presence.	23	41.1
Bonded, I felt the therapy dog liked me.	28	50
Very bonded, I felt the therapy dog liked me over other students.	5	8.9
Did the inclusion of the therapy dog in learning activities influence your mood?		
Definitely	47	83.9
Somewhat	7	12.5
Neutral	1	1.8
Not at all	1	1.8
Did the presence of the therapy dog assist to take your mind off your stressors?		
Definitely	37	66.1
Somewhat	13	23.2
Neutral	5	8.9
Not at all	1	1.8
Did the presence of the therapy dog motivate you to attend learning activities?		
Definitely	39	69.6
Somewhat	10	17.9
Neutral	5	8.9
Not at all	2	3.6
Did the presence of the therapy dog influence your engagement within learning activities?		
Definitely	34	60.7
Somewhat	14	25
Neutral	7	12.5
Not at all	1	1.8
Did the presence of the therapy dog improve your overall learning experience?		
Definitely	38	67.9
Somewhat	15	26.8
Neutral	2	3.6
Not at all	1	1.8
* Were there times throughout the semester where you found the inclusion of the therapy dog most beneficial?		
Throughout the whole semester	35	62.5
When assessments were due	8	14.3
Periods of time within the semester when I was having a difficult time in my life	6	10.7
Start of the semester	4	7.1
I did not find the inclusion of the therapy dog beneficial	2	3.6
Before exams	1	1.8
How would you rate your overall experience of animal-assisted education?		
Extremely satisfied	37	66.1
Satisfied	15	26.8
Neutral	4	7.1
Would you recommend that universities include animal-assisted education into student learning activities? *n* = 54		
Yes	53	98.1
No	1	1.9

* Multiple response question.

## Data Availability

Data collected during this study may be obtained upon request to the authors.

## References

[1] Bantjes J., Hunt X., Stein D. (2022). Public health approaches to promoting university students’ mental health: A global perspective. Curr. Psychiatry Rep..

[2] Linley W., Dorstyn D., Dorstyn D. (2018). Understanding Australian university students’ mental health help-seeking: An empirical and theoretical investigation. Aust. J. Psychol..

[3] Usher Q., Curran C. (2019). Predicting Australia’s university students’ mental health status. Health Promot. Int..

[4] Pendry P., Kuzara S., Gee N. (2019). Evaluation of undergraduate students’ responsiveness to a 4-week university-based animal-assisted stress prevention program. Int. J. Environ. Res. Public Health.

[5] Gee N., Fine A., Schuck S., Fine A. (2015). Animals in educational settings: Research and practice. Handbook on Animal-Assisted Therapy: Foundations and Guidelines for Animal-Assisted Interventions.

[6] Binder A.J., Parish-Plass N., Kirby M., Winkle M., Skwerer D.P., Ackerman L., Brosig C., Coombe W., Delisle E., Enders-Slegers M.-J. (2024). Recommendations for uniform terminology in animal-assisted services (AAS). Hum. Anim. Interact..

[7] Steel J. (2024). Reading to dogs as a form of animal-assisted education: Are positive outcomes supported by quality research?. Literacy.

[8] Barker S., Barker R., McCain N., Schubert C. (2017). The effect of a canine-assisted activity on college student perceptions of family supports and current stressors. Anthrozoös.

[9] Binfet J., Passmore H. (2016). Hounds and homesickness: The effects of an animal- assisted therapeutic intervention for first year university students. Anthrozoös.

[10] McArthyr A., Syrnyk C. (2018). On-campus animal-assisted therapy events. Soc. Anim..

[11] Rothkoph C., Schworm S. (2021). Exploring dog-assisted interventions in higher education: Students’ attitudes and perceived effects on well-being. Int. J. Environ. Res. Public Health.

[12] Chute A., Vihos J., Johnston S., Buro K., Velupillai N. (2023). The effect of animal-assisted intervention on undergraduate students’ perception of momentary stress. Frontiers.

[13] Pendry P., Carr A., Gee N., Vandagriff J. (2020). Randomised trial examining effects of animal-assisted intervention and stress related symptoms on college students’ learning and study skills. Int. J. Environ. Res. Public Health.

[14] Huber A., Klug S., Abraham A., Westenberg E., Schmidt V., Winkler A. (2022). Animal-assisted interventions improve mental, but not cognitive or physiological health outcomes of higher education students: A systematic review and meta-analysis. Int. J. Ment. Health Addict..

[15] Parbery-Clark C., Lubamba M., Tanner L., McColl E. (2021). Animal-assisted interventions for the improvement of mental health outcomes in higher education students: A systematic review of randomised control trials. Int. J. Environ. Res. Public Health.

[16] Hill J., Mensforth S., Waldby L., Fleming J., Quinlan T., Driscoll C. (2024). Enhancing Occupational Therapists’ Education of Animal-Assisted Therapy: A Students’ Experience.

[17] Bengtsson M. (2016). How to plan and perform a qualitative study using content analysis. Nurs. Plus Open.

[18] Hunt M., Al-Braiki F., Dailey S., Russell R., Simon K. (2018). Mindfulness Training, Yoga, or Both? Dismantling the Active Components of a Mindfulness-Based Stress Reduction Intervention. Mindfulness.

[19] Grajfoner D., Harte E., Potter L.M., McGuigan N. (2017). The Effect of Dog-Assisted Intervention on Student Well-Being, Mood, and Anxiety. Int. J. Environ. Res. Public Health.

[20] Baird R., Berger E., Grové C. (2023). Therapy dogs and school wellbeing: A qualitative study. J. Vet. Behav..

[21] Hill J., Ziviani J., Driscoll C. (2020). Canine assisted occupational therapy: A parents’ perspective. Aust. Occup. Ther. J..

[22] Hill J., Ziviani J., Driscoll C., Cawdell-Smith J. (2019). Canine assisted occupational therapy for children on the autism spectrum: Challenges in practice. Br. J. Occup. Ther..

[23] Winkle M., Jacaruso A. (2023). Embarking on the Journey for Animal Assisted Therapy in OT.

